# Plummer-Vinson Syndrome Presenting With Aspiration Pneumonia: A Case Report

**DOI:** 10.7759/cureus.108736

**Published:** 2026-05-12

**Authors:** Seth I Jamieson, Samadhi H Alagoda, Elsafi Gafer, Ronan Keane, Saddam A Hassan

**Affiliations:** 1 General Medicine, Northern Health and Social Care Trust, Belfast, GBR; 2 General Medicine, Exeter Hospital, Exeter, GBR; 3 Gastroenterology, Daisy Hill Hospital, Newry, GBR; 4 General Medicine, Daisy Hill Hospital, Newry, GBR; 5 Internal Medicine, Daisy Hill Hospital, Newry, GBR

**Keywords:** iron deficiency anemia (ida), oesophageal web, plummer–vinson syndrome, rare cause of dysphagia, recurrent aspiration pneumonia

## Abstract

Plummer-Vinson syndrome (PVS) is a rare condition characterised by the triad of iron deficiency anaemia, dysphagia, and oesophageal webs. While dysphagia is a recognised feature, complications such as aspiration are infrequently reported. We describe a 60-year-old woman with a history of Crohn’s disease who presented with aspiration pneumonia in the context of previously undiagnosed PVS. This case demonstrates aspiration pneumonia as a potential and under-recognised complication of proximal oesophageal webs and highlights the importance of early investigation of dysphagia in patients with iron deficiency.

## Introduction

Plummer-Vinson syndrome is defined by the coexistence of iron deficiency anaemia, dysphagia, and proximal oesophageal web formation and is predominantly seen in middle-aged women [1]. Although its incidence has declined in developed countries, it remains an important differential diagnosis in patients presenting with dysphagia and biochemical evidence of iron deficiency [1-5]. Complications arising from impaired bolus transit are poorly described and whilst aspiration has been described, aspiration pneumonia as a presenting complaint remains rarely reported and likely under-recognised in the literature [6]. We report a case of PVS presenting with aspiration pneumonia, emphasising the importance of early recognition and appropriate investigation. 

## Case presentation

A 60-year-old woman presented with a three-day history of shortness of breath and a productive cough of green sputum. She attended the emergency department due to acute pleuritic chest pain over the previous 12 hours. She reported a one-year history of progressive dysphagia, predominantly affecting solid foods, with a sensation of food lodging in the throat. These episodes were frequently associated with coughing and regurgitation of undigested material, but she denied any history of chest pain or shortness of breath following dysphagia. Overall, this represents a one-year history of progressive dysphagia culminating in an acute respiratory presentation, which required hospital admission.

Her medical history included Crohn’s disease requiring multiple bowel resections and the formation of an end ileostomy. She had also previously undergone treatment for oestrogen receptor-positive, HER2-negative breast cancer with mastectomy and reconstruction. There was a reported family history of dysphagia affecting her father and paternal grandfather, the significance of which is unclear.

On examination, the patient was tachypneic with reduced oxygen saturation (91% on room air). Inspiratory stridor was noted. Food particles were present in expectorated sputum. Chest auscultation revealed right-sided crepitations, with no other significant findings on systemic examination.

Initial laboratory investigations demonstrated anaemia with features consistent with iron deficiency (Table 1). Although ferritin was within the normal range, this may reflect an acute phase response in the context of infection, with low transferrin saturation supporting iron deficiency. Inflammatory markers were elevated.

**Table 1:** Laboratory results compared to reference ranges

Parameter	Result	Reference Range	Units
Haemoglobin	111	120-160	g/L
Serum iron	4	10-30	µmol/L
Transferrin saturation	6.6	20-50	%
Ferritin	57	15-150	µg/L
C-reactive protein	111	<5	mg/L
White cell count	13.3	4-11	×10⁹/L

Chest radiography demonstrated right middle and lower zone consolidation consistent with aspiration pneumonia.

The patient was commenced on intravenous antibiotics (amoxicillin and clarithromycin), supplemental oxygen therapy and regular chest physiotherapy with good clinical response. She was reviewed by the speech and language therapy team, who reported normal movement of the food bolus in the pharynx and raised concerns of an oesophageal cause of her dysphagia. 

Therefore, an inpatient upper gastrointestinal endoscopy was performed. This revealed a proximal oesophageal web at approximately the C4 vertebral level. Visualisation and therapeutic intervention were limited by the lesion’s proximal location. Biopsies were obtained, which subsequently grew *Candida albicans*, likely representing secondary colonisation in the setting of oesophageal stasis, given she was deemed to be immunocompetent. Antifungal therapy was initiated.

**Figure 1:**
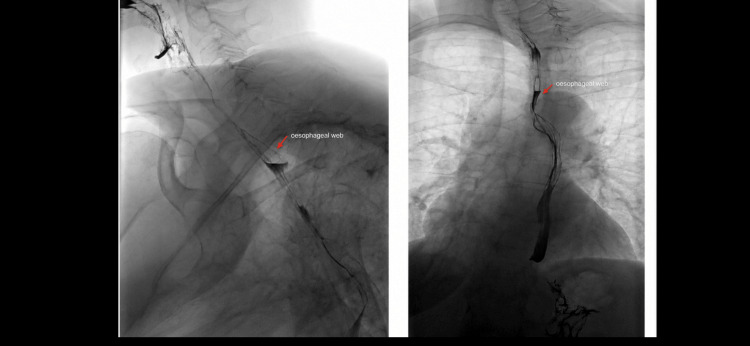
Lateral (left) and AP (right) fluoroscopic barium swallow images demonstrating a proximal cervical oesophageal web at the level of C4 with focal luminal narrowing

A barium swallow study confirmed a circumferential oesophageal web at the level of C4 with moderate luminal narrowing and no complete obstruction (Figure 1). A videofluoroscopic swallow study was unfortunately not available during admission.

Following clinical improvement, the patient was discharged with urgent outpatient follow-up for consideration of endoscopic dilation within six weeks due to the risk of further aspiration events. Oral iron replacement therapy was initiated, with repeat biochemical investigations planned prior to clinic review. She was given safety netting advice regarding safe swallowing and dietary options whilst awaiting definitive treatment. 

## Discussion

Plummer-Vinson syndrome is defined by the triad of iron deficiency anaemia, dysphagia, and oesophageal webs [1]. Although once more prevalent, its incidence has declined significantly in developed countries, likely due to improved nutritional status and earlier detection of iron deficiency [2].

This case illustrates a classical presentation of PVS in a patient with a clear predisposing factor for iron deficiency, namely Crohn’s disease with prior bowel resections. Chronic gastrointestinal blood loss, reduced iron absorption, and nutritional deficiency may have resulted in iron depletion for this patient. Long-term iron deficiency is thought to contribute to mucosal atrophy and web formation, although the exact pathophysiology remains incompletely understood [2].

While dysphagia is a well-recognised feature of PVS, complications such as aspiration are rarely emphasised [3,4]. In this case, the proximal oesophageal web likely led to impaired bolus transit, resulting in retention of food within the upper oesophagus. This predisposes to regurgitation and subsequent aspiration [3]. The location of the web likely increased aspiration risk due to the close proximity to the laryngeal inlet. Recurrent retention of food material within the cervical oesophagus may have promoted aspiration over time, contributing to the development of pneumonia.

The isolation of *Candida albicans* on biopsy likely reflects secondary colonisation due to oesophageal stasis rather than primary pathology, though it may have contributed to symptom severity. This case highlights that early involvement of gastroenterology, speech and language therapists, radiology and dietitians may help reduce morbidity in patients presenting with complex dysphagia and aspiration risk. 

The patient’s family history of dysphagia raises the possibility of genetic or shared environmental contributions, although this association is not well established and warrants further investigation [5]. Differential diagnoses considered included oesophageal malignancy, benign oesophageal stricture, and oropharyngeal dysfunction, but the presence of a web in the context of iron deficiency anaemia strongly supported the diagnosis of PVS.

Recognition of PVS is clinically important not only for symptom relief but also due to its association with an increased risk of upper gastrointestinal malignancy, particularly squamous cell carcinoma of the oesophagus and pharynx [1]. Early diagnosis and management, including iron repletion and endoscopic intervention where indicated, may reduce both morbidity and long-term complications. Although aspiration has been described in PVS, reports of aspiration pneumonia as a presenting manifestation remain limited in the literature. This case highlights that screening high-risk patients for iron deficiency anaemia may reduce the likelihood of developing life-threatening infections such as aspiration pneumonia secondary to oesophageal web formation.

## Conclusions

This case highlights aspiration pneumonia as a potential and under-recognised complication of Plummer-Vinson syndrome. Clinicians should maintain a high index of suspicion for PVS in patients presenting with dysphagia and iron deficiency anaemia, particularly in those with underlying risk factors such as inflammatory bowel disease. Prompt investigation, multidisciplinary assessment, and management are essential to improve patient outcomes and prevent serious complications. This is a single case report and thus requires further research to better characterise any correlation between PVS and aspiration pneumonia.
